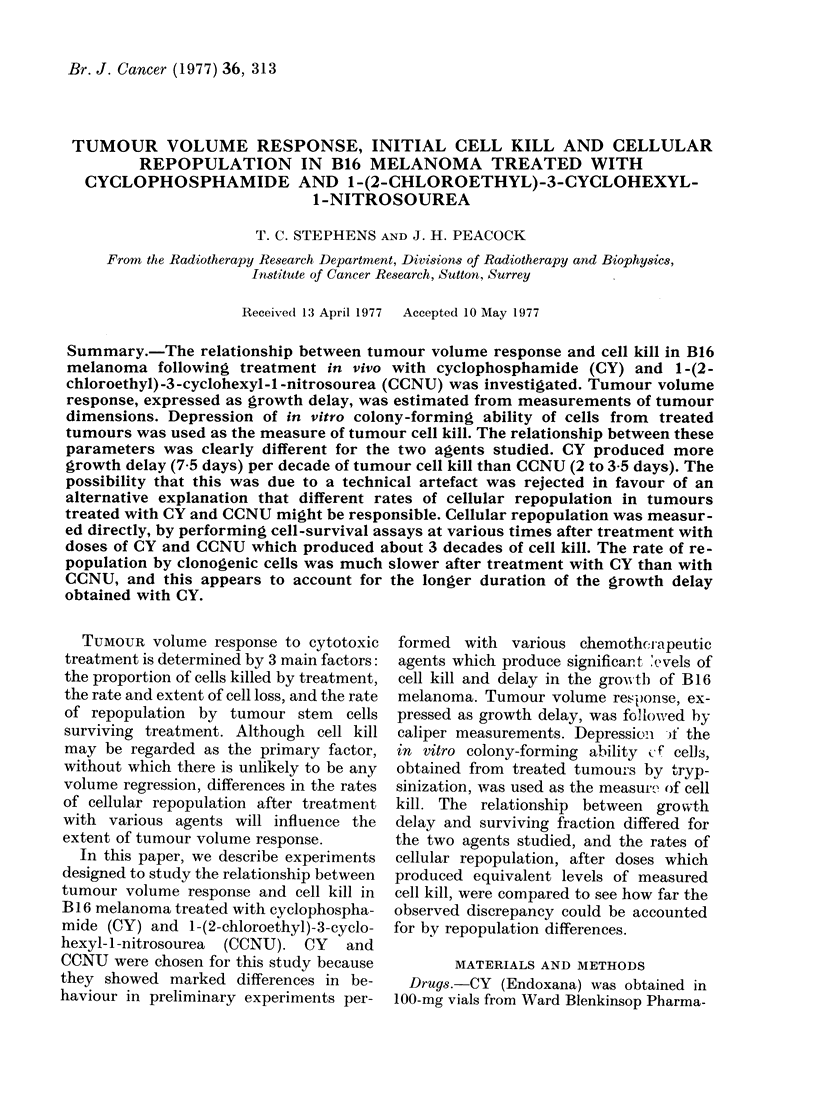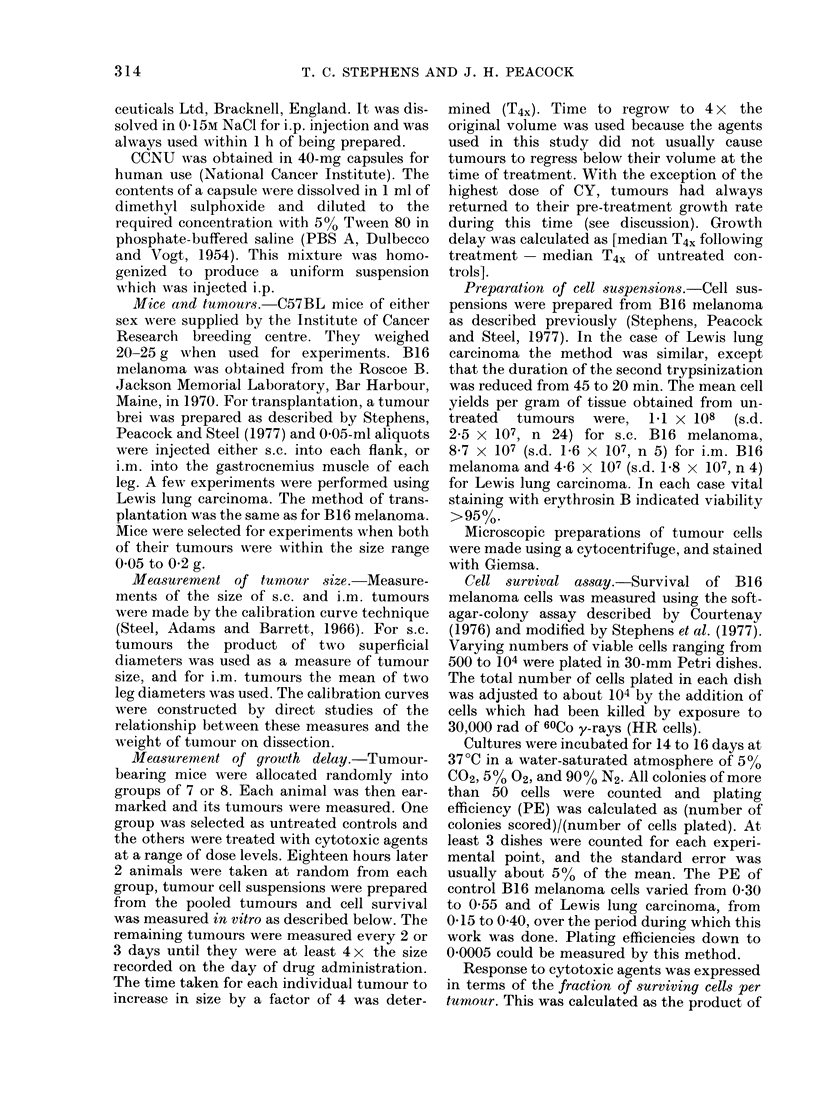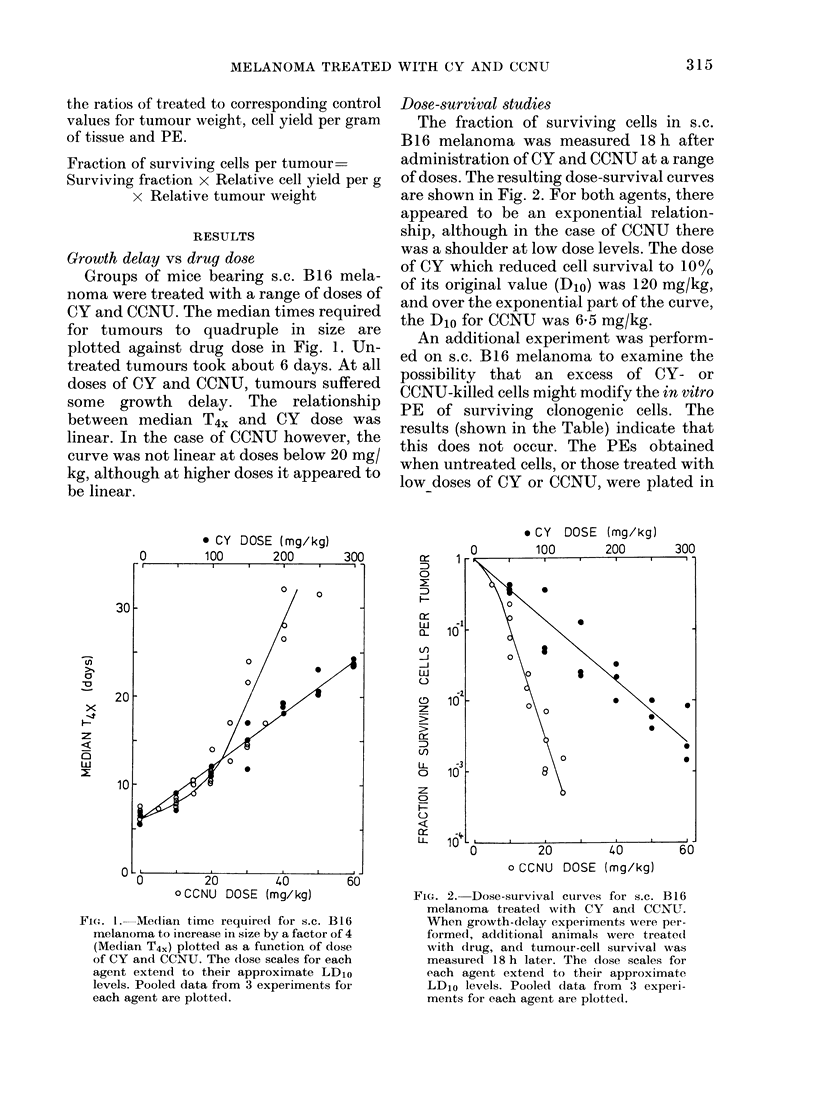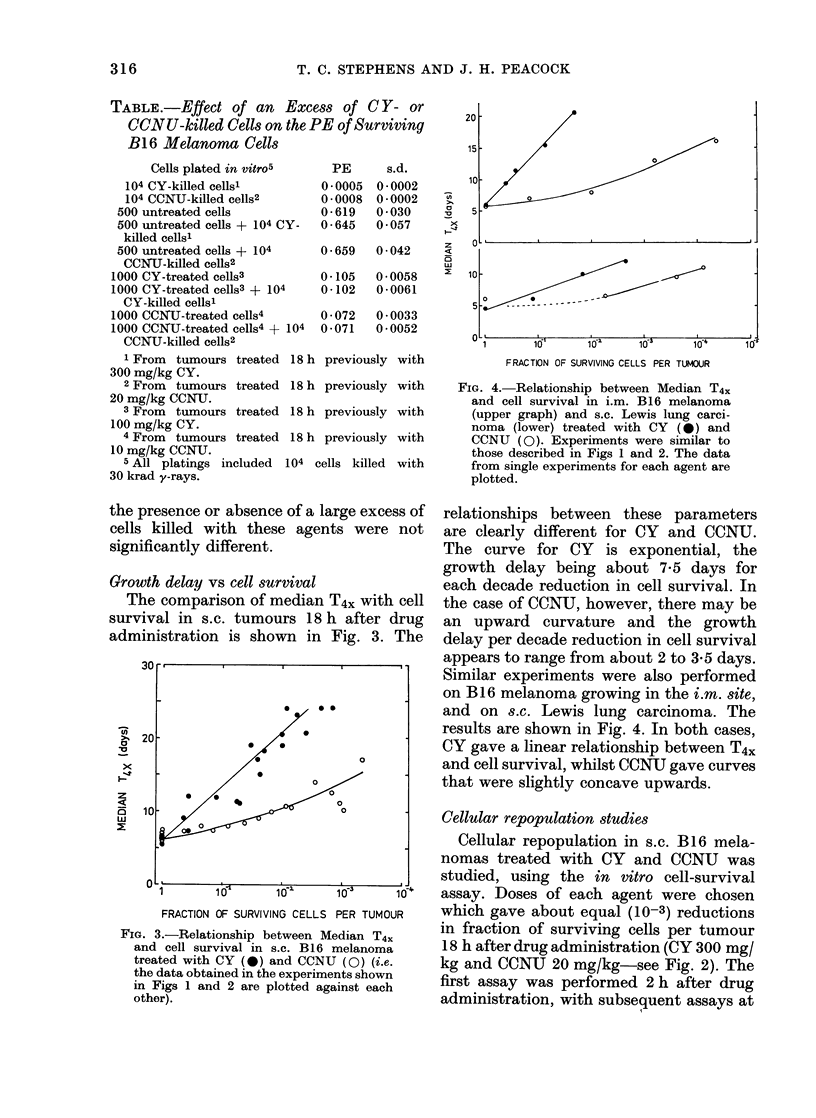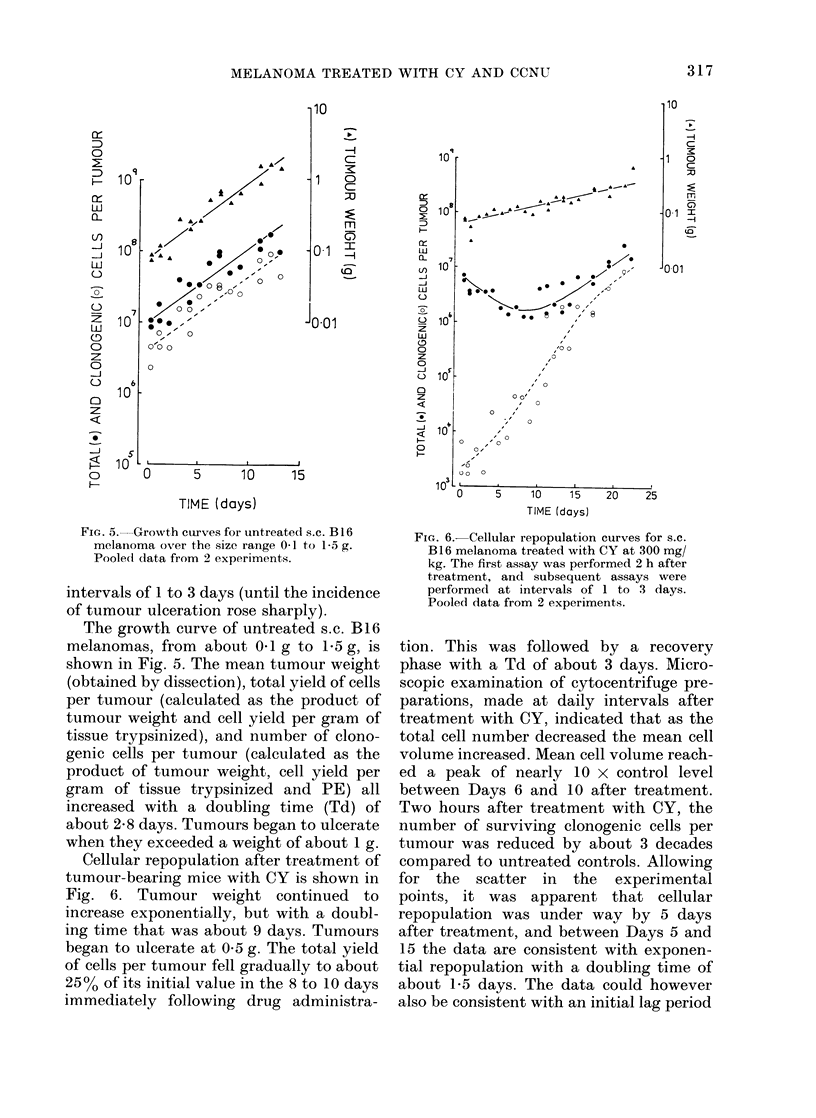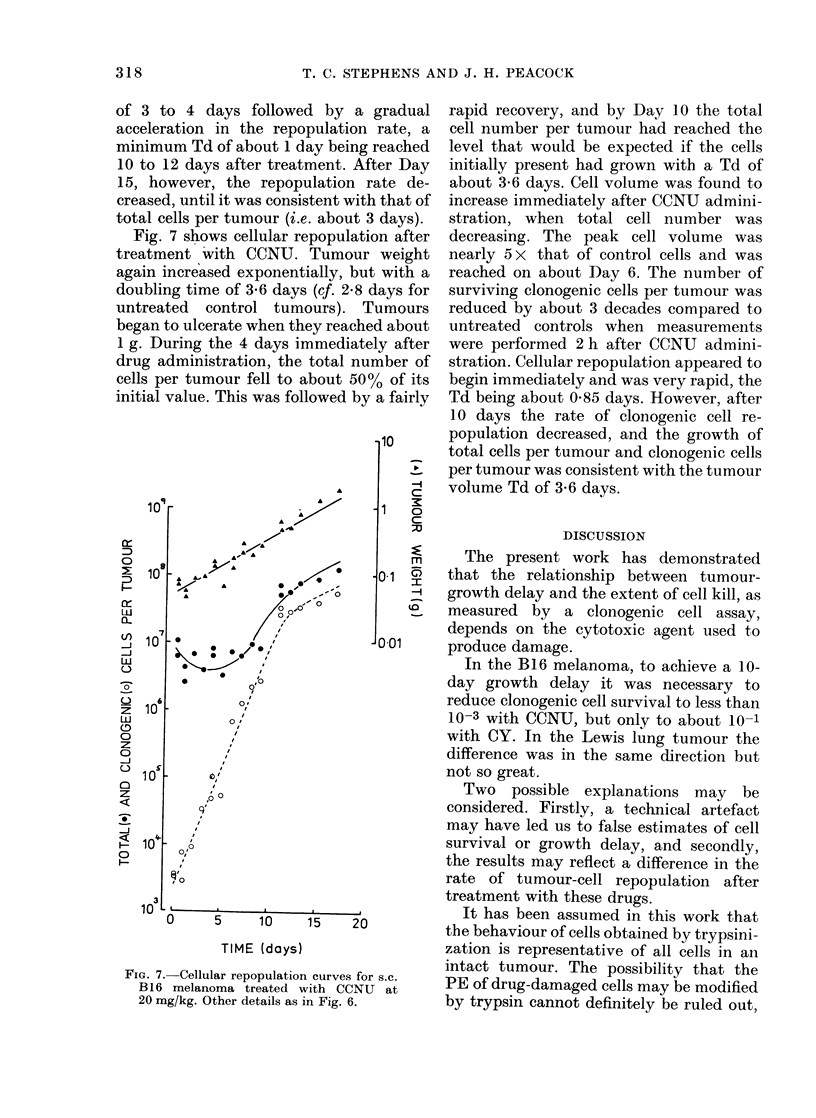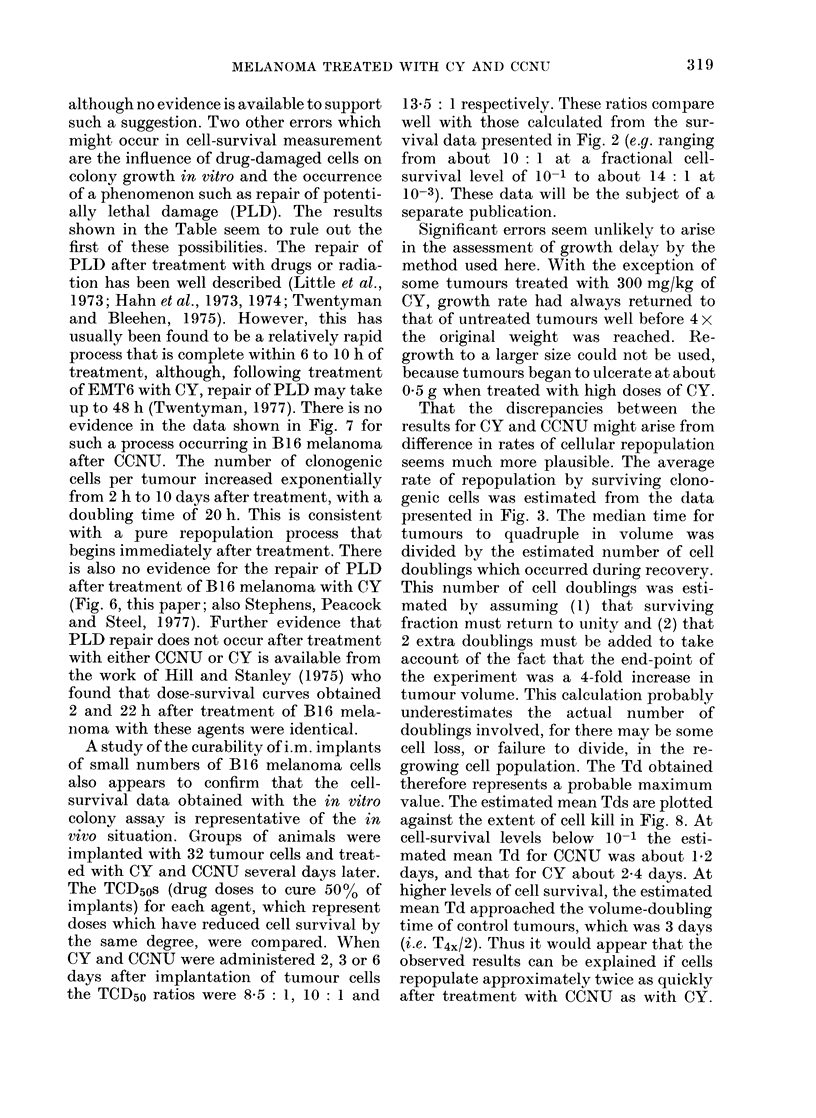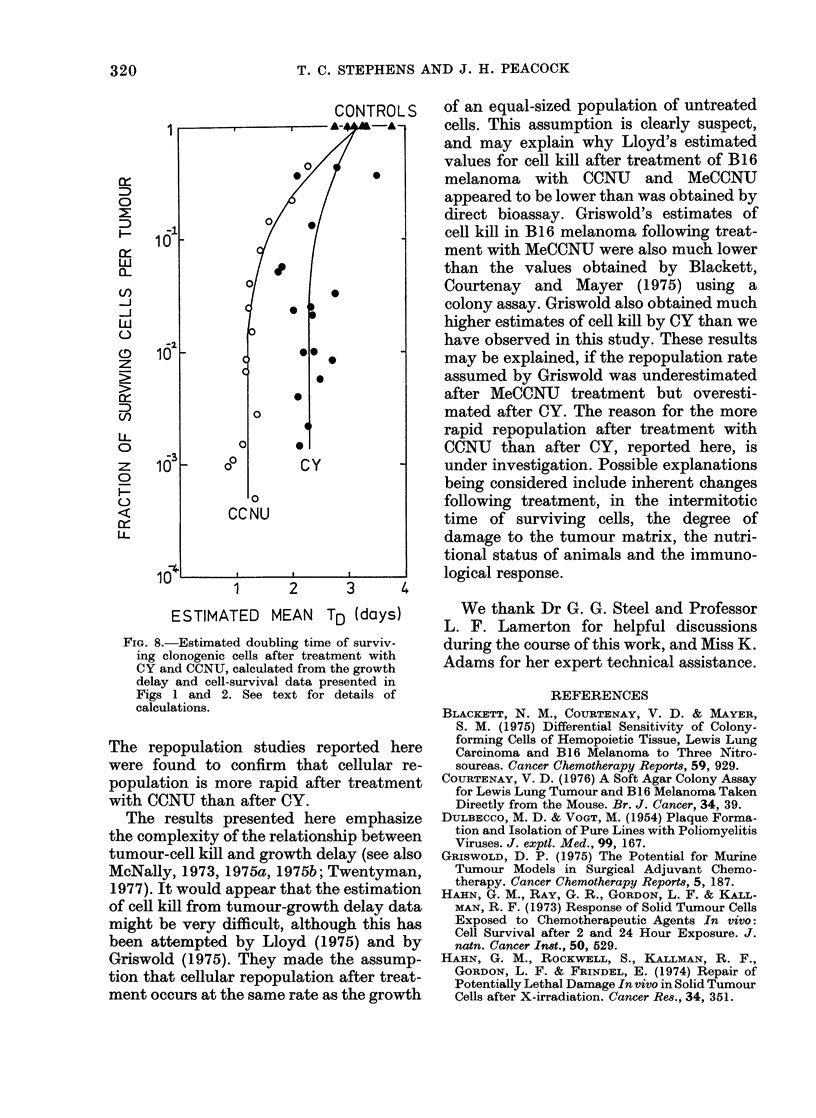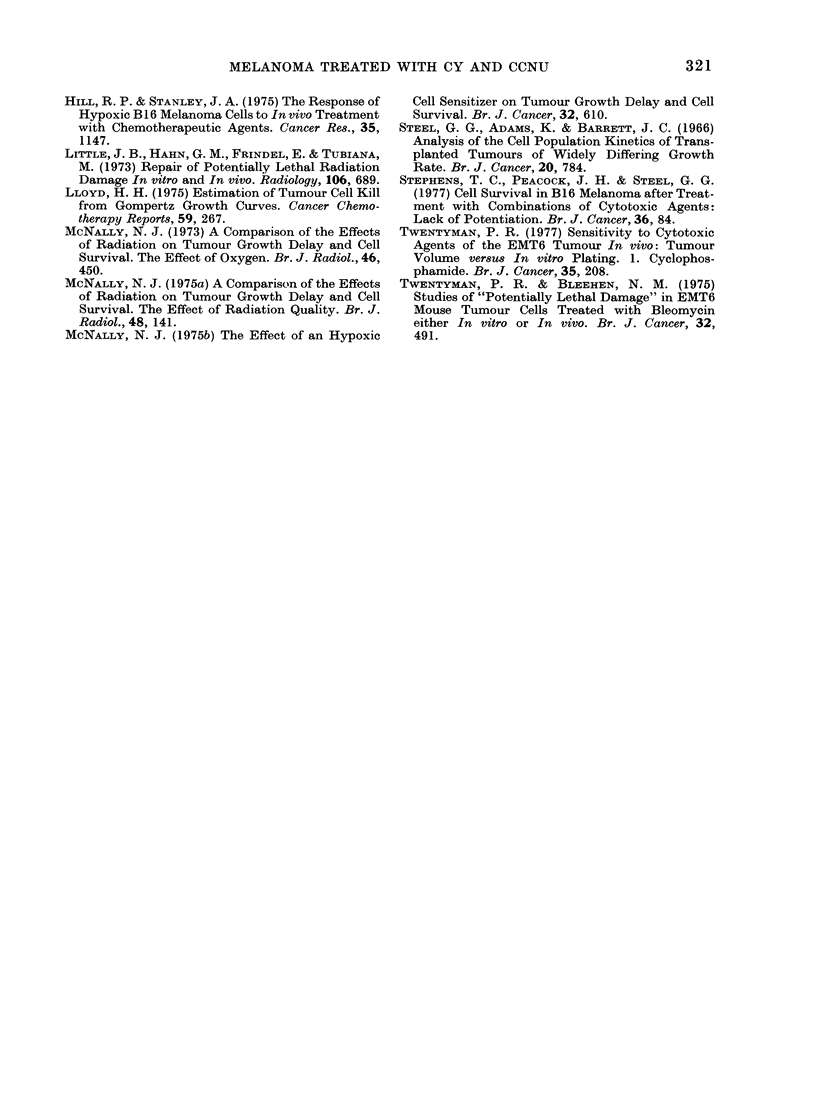# Tumour volume response, initial cell kill and cellular repopulation in B16 melanoma treated with cyclophosphamide and 1-(2-chloroethyl)-3-cyclohexyl-1-nitrosourea.

**DOI:** 10.1038/bjc.1977.195

**Published:** 1977-09

**Authors:** T. C. Stephens, J. H. Peacock

## Abstract

The relationship between tumour volume response and cell kill in B16 melanoma following treatment in vivo with cyclophosphamide (CY) and 1-(2-chloroethyl)-3-cyclohexyl-1-nitrosourea (CCNU) was investigated. Tumour volume response, expressed as growth delay, was estimated from measurements of tumour dimensions. Depression of in vitro colony-forming ability of cells from treated tumours was used as the measure of tumour cell kill. The relationship between these parameters was clearly different for the two agents studied. CY produced more growth delay (7.5 days) per decade of tumour cell kill than CCNU (2 to 3.5 days). The possibility that this was due to a technical artefact was rejected in favour of an alternative explanation that different rates of cellular repopulation in tumours treated with CY and CCNU might be responsible. Cellular repopulation was measured directly, by performing cell-survival assays at various times after treatment with doses of CY and CCNU which produced about 3 decades of cell kill. The rate of repopulation by clonogenic cells was much slower after treatment with CY than with CCNU, and this appears to account for the longer duration of the growth delay obtained with CY.


*Br. J. Cancer* (1977) **36**, 313

### TUMOUR VOLUME RESPONSE, INITIAL CELL KILL AND CELLULAR REPOPULATION IN B16 MELANOMA TREATED WITH CYCLOPHOSPHAMIDE AND 1-(2-CHLOROETHYL)-3-CYCLOHEXYL-1-NITROSOUREA

T. C. STEPHENS AND J. H. PEACOCK

*From the Radiotherapy Research Department, Divisions of Radiotherapy and Biophysics, Institute of Cancer Research, Sutton, Surrey*

Received 13 April 1977 Accepted 10 May 1977

**Summary.—The relationship between tumour volume response and cell kill in B16 melanoma following treatment *in vivo* with cyclophosphamide (CY) and 1-(2-chloroethyl)-3-cyclohexyl-1-nitrosourea (CCNU) was investigated. Tumour volume response, expressed as growth delay, was estimated from measurements of tumour dimensions. Depression of *in vitro* colony-forming ability of cells from treated tumours was used as the measure of tumour cell kill. The relationship between these parameters was clearly different for the two agents studied. CY produced more growth delay (7·5 days) per decade of tumour cell kill than CCNU (2 to 3·5 days). The possibility that this was due to a technical artefact was rejected in favour of an alternative explanation that different rates of cellular repopulation in tumours treated with CY and CCNU might be responsible. Cellular repopulation was measured directly, by performing cell-survival assays at various times after treatment with doses of CY and CCNU which produced about 3 decades of cell kill. The rate of repopulation by clonogenic cells was much slower after treatment with CY than with CCNU, and this appears to account for the longer duration of the growth delay obtained with CY.**

TUMOUR volume response to cytotoxic treatment is determined by 3 main factors: the proportion of cells killed by treatment, the rate and extent of cell loss, and the rate of repopulation by tumour stem cells surviving treatment. Although cell kill may be regarded as the primary factor, without which there is unlikely to be any volume regression, differences in the rates of cellular repopulation after treatment with various agents will influence the extent of tumour volume response.

In this paper, we describe experiments designed to study the relationship between tumour volume response and cell kill in B16 melanoma treated with cyclophosphamide (CY) and 1-(2-chloroethyl)-3-cyclohexyl-1-nitrosourea (CCNU). CY and CCNU were chosen for this study because they showed marked differences in behaviour in preliminary experiments performed with various chemotherapeutic agents which produce significant levels of cell kill and delay in the growth of B16 melanoma. Tumour volume response, expressed as growth delay, was followed by caliper measurements. Depression of the *in vitro* colony-forming ability of cells, obtained from treated tumours by trypsinization, was used as the measure of cell kill. The relationship between growth delay and surviving fraction differed for the two agents studied, and the rates of cellular repopulation, after doses which produced equivalent levels of measured cell kill, were compared to see how far the observed discrepancy could be accounted for by repopulation differences.

### MATERIALS AND METHODS

*Drugs.*—CY (Endoxana) was obtained in 100-mg vials from Ward Blenkinsop Pharmaceuticals Ltd, Bracknell, England. It was dissolved in 0·15M NaCl for i.p. injection and was always used within 1 h of being prepared.

CCNU was obtained in 40-mg capsules for human use (National Cancer Institute). The contents of a capsule were dissolved in 1 ml of dimethyl sulphoxide and diluted to the required concentration with 5% Tween 80 in phosphate-buffered saline (PBS A, Dulbecco and Vogt, 1954). This mixture was homogenized to produce a uniform suspension which was injected i.p.

*Mice and tumours.*—C57BL mice of either sex were supplied by the Institute of Cancer Research breeding centre. They weighed 20–25 g when used for experiments. B16 melanoma was obtained from the Roscoe B. Jackson Memorial Laboratory, Bar Harbour, Maine, in 1970. For transplantation, a tumour brei was prepared as described by Stephens, Peacock and Steel (1977) and 0·05-ml aliquots were injected either s.c. into each flank, or i.m. into the gastrocnemius muscle of each leg. A few experiments were performed using Lewis lung carcinoma. The method of transplantation was the same as for B16 melanoma. Mice were selected for experiments when both of their tumours were within the size range 0·05 to 0·2 g.

*Measurement of tumour size.*—Measurements of the size of s.c. and i.m. tumours were made by the calibration curve technique (Steel, Adams and Barrett, 1966). For s.c. tumours the product of two superficial diameters was used as a measure of tumour size, and for i.m. tumours the mean of two leg diameters was used. The calibration curves were constructed by direct studies of the relationship between these measures and the weight of tumour on dissection.

*Measurement of growth delay.*—Tumour-bearing mice were allocated randomly into groups of 7 or 8. Each animal was then ear-marked and its tumours were measured. One group was selected as untreated controls and the others were treated with cytotoxic agents at a range of dose levels. Eighteen hours later 2 animals were taken at random from each group, tumour cell suspensions were prepared from the pooled tumours and cell survival was measured *in vitro* as described below. The remaining tumours were measured every 2 or 3 days until they were at least 4× the size recorded on the day of drug administration. The time taken for each individual tumour to increase in size by a factor of 4 was determined ($T_{4x}$). Time to regrow to 4× the original volume was used because the agents used in this study did not usually cause tumours to regress below their volume at the time of treatment. With the exception of the highest dose of CY, tumours had always returned to their pre-treatment growth rate during this time (see discussion). Growth delay was calculated as [median $T_{4x}$ following treatment − median $T_{4x}$ of untreated controls].

*Preparation of cell suspensions.*—Cell suspensions were prepared from B16 melanoma as described previously (Stephens, Peacock and Steel, 1977). In the case of Lewis lung carcinoma the method was similar, except that the duration of the second trypsinization was reduced from 45 to 20 min. The mean cell yields per gram of tissue obtained from untreated tumours were, $1{\cdot}1 \times 10^8$ (s.d. $2{\cdot}5 \times 10^7$, n 24) for s.c. B16 melanoma, $8{\cdot}7 \times 10^7$ (s.d. $1{\cdot}6 \times 10^7$, n 5) for i.m. B16 melanoma and $4{\cdot}6 \times 10^7$ (s.d. $1{\cdot}8 \times 10^7$, n 4) for Lewis lung carcinoma. In each case vital staining with erythrosin B indicated viability >95%.

Microscopic preparations of tumour cells were made using a cytocentrifuge, and stained with Giemsa.

*Cell survival assay.*—Survival of B16 melanoma cells was measured using the soft-agar-colony assay described by Courtenay (1976) and modified by Stephens *et al.* (1977). Varying numbers of viable cells ranging from 500 to $10^4$ were plated in 30-mm Petri dishes. The total number of cells plated in each dish was adjusted to about $10^4$ by the addition of cells which had been killed by exposure to 30,000 rad of $^{60}$Co $\gamma$-rays (HR cells).

Cultures were incubated for 14 to 16 days at 37 °C in a water-saturated atmosphere of 5% $CO_2$, 5% $O_2$, and 90% $N_2$. All colonies of more than 50 cells were counted and plating efficiency (PE) was calculated as (number of colonies scored)/(number of cells plated). At least 3 dishes were counted for each experimental point, and the standard error was usually about 5% of the mean. The PE of control B16 melanoma cells varied from 0·30 to 0·55 and of Lewis lung carcinoma, from 0·15 to 0·40, over the period during which this work was done. Plating efficiencies down to 0·0005 could be measured by this method.

Response to cytotoxic agents was expressed in terms of the *fraction of surviving cells per tumour*. This was calculated as the product of the ratios of treated to corresponding control values for tumour weight, cell yield per gram of tissue and PE.

Fraction of surviving cells per tumour= Surviving fraction × Relative cell yield per g × Relative tumour weight

### RESULTS

*Growth delay* vs *drug dose*

Groups of mice bearing s.c. B16 melanoma were treated with a range of doses of CY and CCNU. The median times required for tumours to quadruple in size are plotted against drug dose in Fig. 1. Untreated tumours took about 6 days. At all doses of CY and CCNU, tumours suffered some growth delay. The relationship between median $T_{4x}$ and CY dose was linear. In the case of CCNU however, the curve was not linear at doses below 20 mg/kg, although at higher doses it appeared to be linear.

*Dose-survival studies*

The fraction of surviving cells in s.c. B16 melanoma was measured 18 h after administration of CY and CCNU at a range of doses. The resulting dose-survival curves are shown in Fig. 2. For both agents, there appeared to be an exponential relationship, although in the case of CCNU there was a shoulder at low dose levels. The dose of CY which reduced cell survival to 10% of its original value ($D_{10}$) was 120 mg/kg, and over the exponential part of the curve, the $D_{10}$ for CCNU was 6·5 mg/kg.

An additional experiment was performed on s.c. B16 melanoma to examine the possibility that an excess of CY- or CCNU-killed cells might modify the *in vitro* PE of surviving clonogenic cells. The results (shown in the Table) indicate that this does not occur. The PEs obtained when untreated cells, or those treated with low doses of CY or CCNU, were plated in

FIG. 1.—Median time required for s.c. B16 melanoma to increase in size by a factor of 4 (Median $T_{4x}$) plotted as a function of dose of CY and CCNU. The dose scales for each agent extend to their approximate $LD_{10}$ levels. Pooled data from 3 experiments for each agent are plotted.

FIG. 2.—Dose-survival curves for s.c. B16 melanoma treated with CY and CCNU. When growth-delay experiments were performed, additional animals were treated with drug, and tumour-cell survival was measured 18 h later. The dose scales for each agent extend to their approximate $LD_{10}$ levels. Pooled data from 3 experiments for each agent are plotted.

TABLE.—*Effect of an Excess of CY- or CCNU-killed Cells on the PE of Surviving B16 Melanoma Cells*

| Cells plated *in vitro*[5] | PE | s.d. |
|---|---|---|
| $10^4$ CY-killed cells[1] | 0·0005 | 0·0002 |
| $10^4$ CCNU-killed cells[2] | 0·0008 | 0·0002 |
| 500 untreated cells | 0·619 | 0·030 |
| 500 untreated cells + $10^4$ CY-killed cells[1] | 0·645 | 0·057 |
| 500 untreated cells + $10^4$ CCNU-killed cells[2] | 0·659 | 0·042 |
| 1000 CY-treated cells[3] | 0·105 | 0·0058 |
| 1000 CY-treated cells[3] + $10^4$ CY-killed cells[1] | 0·102 | 0·0061 |
| 1000 CCNU-treated cells[4] | 0·072 | 0·0033 |
| 1000 CCNU-treated cells[4] + $10^4$ CCNU-killed cells[2] | 0·071 | 0·0052 |

[1] From tumours treated 18 h previously with 300 mg/kg CY.
[2] From tumours treated 18 h previously with 20 mg/kg CCNU.
[3] From tumours treated 18 h previously with 100 mg/kg CY.
[4] From tumours treated 18 h previously with 10 mg/kg CCNU.
[5] All platings included $10^4$ cells killed with 30 krad $\gamma$-rays.

the presence or absence of a large excess of cells killed with these agents were not significantly different.

### *Growth delay* vs *cell survival*

The comparison of median $T_{4x}$ with cell survival in s.c. tumours 18 h after drug administration is shown in Fig. 3. The

FIG. 3.—Relationship between Median $T_{4x}$ and cell survival in s.c. B16 melanoma treated with CY (●) and CCNU (○) (*i.e.* the data obtained in the experiments shown in Figs 1 and 2 are plotted against each other).

FIG. 4.—Relationship between Median $T_{4x}$ and cell survival in i.m. B16 melanoma (upper graph) and s.c. Lewis lung carcinoma (lower) treated with CY (●) and CCNU (○). Experiments were similar to those described in Figs 1 and 2. The data from single experiments for each agent are plotted.

relationships between these parameters are clearly different for CY and CCNU. The curve for CY is exponential, the growth delay being about 7·5 days for each decade reduction in cell survival. In the case of CCNU, however, there may be an upward curvature and the growth delay per decade reduction in cell survival appears to range from about 2 to 3·5 days. Similar experiments were also performed on B16 melanoma growing in the *i.m. site*, and on *s.c.* Lewis lung carcinoma. The results are shown in Fig. 4. In both cases, CY gave a linear relationship between $T_{4x}$ and cell survival, whilst CCNU gave curves that were slightly concave upwards.

### *Cellular repopulation studies*

Cellular repopulation in s.c. B16 melanomas treated with CY and CCNU was studied, using the *in vitro* cell-survival assay. Doses of each agent were chosen which gave about equal ($10^{-3}$) reductions in fraction of surviving cells per tumour 18 h after drug administration (CY 300 mg/kg and CCNU 20 mg/kg—see Fig. 2). The first assay was performed 2 h after drug administration, with subsequent assays at

Fig. 5.—Growth curves for untreated s.c. B16 melanoma over the size range 0·1 to 1·5 g. Pooled data from 2 experiments.

Fig. 6.—Cellular repopulation curves for s.c. B16 melanoma treated with CY at 300 mg/kg. The first assay was performed 2 h after treatment, and subsequent assays were performed at intervals of 1 to 3 days. Pooled data from 2 experiments.

intervals of 1 to 3 days (until the incidence of tumour ulceration rose sharply).

The growth curve of untreated s.c. B16 melanomas, from about 0·1 g to 1·5 g, is shown in Fig. 5. The mean tumour weight (obtained by dissection), total yield of cells per tumour (calculated as the product of tumour weight and cell yield per gram of tissue trypsinized), and number of clonogenic cells per tumour (calculated as the product of tumour weight, cell yield per gram of tissue trypsinized and PE) all increased with a doubling time (Td) of about 2·8 days. Tumours began to ulcerate when they exceeded a weight of about 1 g.

Cellular repopulation after treatment of tumour-bearing mice with CY is shown in Fig. 6. Tumour weight continued to increase exponentially, but with a doubling time that was about 9 days. Tumours began to ulcerate at 0·5 g. The total yield of cells per tumour fell gradually to about 25% of its initial value in the 8 to 10 days immediately following drug administration. This was followed by a recovery phase with a Td of about 3 days. Microscopic examination of cytocentrifuge preparations, made at daily intervals after treatment with CY, indicated that as the total cell number decreased the mean cell volume increased. Mean cell volume reached a peak of nearly 10 × control level between Days 6 and 10 after treatment. Two hours after treatment with CY, the number of surviving clonogenic cells per tumour was reduced by about 3 decades compared to untreated controls. Allowing for the scatter in the experimental points, it was apparent that cellular repopulation was under way by 5 days after treatment, and between Days 5 and 15 the data are consistent with exponential repopulation with a doubling time of about 1·5 days. The data could however also be consistent with an initial lag period of 3 to 4 days followed by a gradual acceleration in the repopulation rate, a minimum Td of about 1 day being reached 10 to 12 days after treatment. After Day 15, however, the repopulation rate decreased, until it was consistent with that of total cells per tumour (*i.e.* about 3 days).

Fig. 7 shows cellular repopulation after treatment with CCNU. Tumour weight again increased exponentially, but with a doubling time of 3·6 days (*cf.* 2·8 days for untreated control tumours). Tumours began to ulcerate when they reached about 1 g. During the 4 days immediately after drug administration, the total number of cells per tumour fell to about 50% of its initial value. This was followed by a fairly rapid recovery, and by Day 10 the total cell number per tumour had reached the level that would be expected if the cells initially present had grown with a Td of about 3·6 days. Cell volume was found to increase immediately after CCNU administration, when total cell number was decreasing. The peak cell volume was nearly 5× that of control cells and was reached on about Day 6. The number of surviving clonogenic cells per tumour was reduced by about 3 decades compared to untreated controls when measurements were performed 2 h after CCNU administration. Cellular repopulation appeared to begin immediately and was very rapid, the Td being about 0·85 days. However, after 10 days the rate of clonogenic cell repopulation decreased, and the growth of total cells per tumour and clonogenic cells per tumour was consistent with the tumour volume Td of 3·6 days.

FIG. 7.—Cellular repopulation curves for s.c. B16 melanoma treated with CCNU at 20 mg/kg. Other details as in Fig. 6.

### DISCUSSION

The present work has demonstrated that the relationship between tumour-growth delay and the extent of cell kill, as measured by a clonogenic cell assay, depends on the cytotoxic agent used to produce damage.

In the B16 melanoma, to achieve a 10-day growth delay it was necessary to reduce clonogenic cell survival to less than $10^{-3}$ with CCNU, but only to about $10^{-1}$ with CY. In the Lewis lung tumour the difference was in the same direction but not so great.

Two possible explanations may be considered. Firstly, a technical artefact may have led us to false estimates of cell survival or growth delay, and secondly, the results may reflect a difference in the rate of tumour-cell repopulation after treatment with these drugs.

It has been assumed in this work that the behaviour of cells obtained by trypsinization is representative of all cells in an intact tumour. The possibility that the PE of drug-damaged cells may be modified by trypsin cannot definitely be ruled out, although no evidence is available to support such a suggestion. Two other errors which might occur in cell-survival measurement are the influence of drug-damaged cells on colony growth *in vitro* and the occurrence of a phenomenon such as repair of potentially lethal damage (PLD). The results shown in the Table seem to rule out the first of these possibilities. The repair of PLD after treatment with drugs or radiation has been well described (Little *et al.*, 1973; Hahn *et al.*, 1973, 1974; Twentyman and Bleehen, 1975). However, this has usually been found to be a relatively rapid process that is complete within 6 to 10 h of treatment, although, following treatment of EMT6 with CY, repair of PLD may take up to 48 h (Twentyman, 1977). There is no evidence in the data shown in Fig. 7 for such a process occurring in B16 melanoma after CCNU. The number of clonogenic cells per tumour increased exponentially from 2 h to 10 days after treatment, with a doubling time of 20 h. This is consistent with a pure repopulation process that begins immediately after treatment. There is also no evidence for the repair of PLD after treatment of B16 melanoma with CY (Fig. 6, this paper; also Stephens, Peacock and Steel, 1977). Further evidence that PLD repair does not occur after treatment with either CCNU or CY is available from the work of Hill and Stanley (1975) who found that dose-survival curves obtained 2 and 22 h after treatment of B16 melanoma with these agents were identical.

A study of the curability of i.m. implants of small numbers of B16 melanoma cells also appears to confirm that the cell-survival data obtained with the *in vitro* colony assay is representative of the *in vivo* situation. Groups of animals were implanted with 32 tumour cells and treated with CY and CCNU several days later. The $TCD_{50}$s (drug doses to cure 50% of implants) for each agent, which represent doses which have reduced cell survival by the same degree, were compared. When CY and CCNU were administered 2, 3 or 6 days after implantation of tumour cells the $TCD_{50}$ ratios were 8·5 : 1, 10 : 1 and 13·5 : 1 respectively. These ratios compare well with those calculated from the survival data presented in Fig. 2 (*e.g.* ranging from about 10 : 1 at a fractional cell-survival level of $10^{-1}$ to about 14 : 1 at $10^{-3}$). These data will be the subject of a separate publication.

Significant errors seem unlikely to arise in the assessment of growth delay by the method used here. With the exception of some tumours treated with 300 mg/kg of CY, growth rate had always returned to that of untreated tumours well before 4× the original weight was reached. Regrowth to a larger size could not be used, because tumours began to ulcerate at about 0·5 g when treated with high doses of CY.

That the discrepancies between the results for CY and CCNU might arise from difference in rates of cellular repopulation seems much more plausible. The average rate of repopulation by surviving clonogenic cells was estimated from the data presented in Fig. 3. The median time for tumours to quadruple in volume was divided by the estimated number of cell doublings which occurred during recovery. This number of cell doublings was estimated by assuming (1) that surviving fraction must return to unity and (2) that 2 extra doublings must be added to take account of the fact that the end-point of the experiment was a 4-fold increase in tumour volume. This calculation probably underestimates the actual number of doublings involved, for there may be some cell loss, or failure to divide, in the regrowing cell population. The Td obtained therefore represents a probable maximum value. The estimated mean Tds are plotted against the extent of cell kill in Fig. 8. At cell-survival levels below $10^{-1}$ the estimated mean Td for CCNU was about 1·2 days, and that for CY about 2·4 days. At higher levels of cell survival, the estimated mean Td approached the volume-doubling time of control tumours, which was 3 days (*i.e.* $T_{4x}/2$). Thus it would appear that the observed results can be explained if cells repopulate approximately twice as quickly after treatment with CCNU as with CY.

FIG. 8.—Estimated doubling time of surviving clonogenic cells after treatment with CY and CCNU, calculated from the growth delay and cell-survival data presented in Figs 1 and 2. See text for details of calculations.

The repopulation studies reported here were found to confirm that cellular repopulation is more rapid after treatment with CCNU than after CY.

The results presented here emphasize the complexity of the relationship between tumour-cell kill and growth delay (see also McNally, 1973, 1975*a*, 1975*b*; Twentyman, 1977). It would appear that the estimation of cell kill from tumour-growth delay data might be very difficult, although this has been attempted by Lloyd (1975) and by Griswold (1975). They made the assumption that cellular repopulation after treatment occurs at the same rate as the growth of an equal-sized population of untreated cells. This assumption is clearly suspect, and may explain why Lloyd's estimated values for cell kill after treatment of B16 melanoma with CCNU and MeCCNU appeared to be lower than was obtained by direct bioassay. Griswold's estimates of cell kill in B16 melanoma following treatment with MeCCNU were also much lower than the values obtained by Blackett, Courtenay and Mayer (1975) using a colony assay. Griswold also obtained much higher estimates of cell kill by CY than we have observed in this study. These results may be explained, if the repopulation rate assumed by Griswold was underestimated after MeCCNU treatment but overestimated after CY. The reason for the more rapid repopulation after treatment with CCNU than after CY, reported here, is under investigation. Possible explanations being considered include inherent changes following treatment, in the intermitotic time of surviving cells, the degree of damage to the tumour matrix, the nutritional status of animals and the immunological response.

We thank Dr G. G. Steel and Professor L. F. Lamerton for helpful discussions during the course of this work, and Miss K. Adams for her expert technical assistance.

### REFERENCES

BLACKETT, N. M., COURTENAY, V. D. & MAYER, S. M. (1975) Differential Sensitivity of Colony-forming Cells of Hemopoietic Tissue, Lewis Lung Carcinoma and B16 Melanoma to Three Nitrosoureas. *Cancer Chemotherapy Reports*, **59**, 929.

COURTENAY, V. D. (1976) A Soft Agar Colony Assay for Lewis Lung Tumour and B16 Melanoma Taken Directly from the Mouse. *Br. J. Cancer*, **34**, 39.

DULBECCO, M. D. & VOGT, M. (1954) Plaque Formation and Isolation of Pure Lines with Poliomyelitis Viruses. *J. exptl. Med.*, **99**, 167.

GRISWOLD, D. P. (1975) The Potential for Murine Tumour Models in Surgical Adjuvant Chemotherapy. *Cancer Chemotherapy Reports*, **5**, 187.

HAHN, G. M., RAY, G. R., GORDON, L. F. & KALLMAN, R. F. (1973) Response of Solid Tumour Cells Exposed to Chemotherapeutic Agents *In vivo*: Cell Survival after 2 and 24 Hour Exposure. *J. natn. Cancer Inst.*, **50**, 529.

HAHN, G. M., ROCKWELL, S., KALLMAN, R. F., GORDON, L. F. & FRINDEL, E. (1974) Repair of Potentially Lethal Damage *In vivo* in Solid Tumour Cells after X-irradiation. *Cancer Res.*, **34**, 351.

HILL, R. P. & STANLEY, J. A. (1975) The Response of Hypoxic B16 Melanoma Cells to *In vivo* Treatment with Chemotherapeutic Agents. *Cancer Res.*, **35**, 1147.

LITTLE, J. B., HAHN, G. M., FRINDEL, E. & TUBIANA, M. (1973) Repair of Potentially Lethal Radiation Damage *In vitro* and *In vivo*. *Radiology*, **106**, 689.

LLOYD, H. H. (1975) Estimation of Tumour Cell Kill from Gompertz Growth Curves. *Cancer Chemotherapy Reports*, **59**, 267.

MCNALLY, N. J. (1973) A Comparison of the Effects of Radiation on Tumour Growth Delay and Cell Survival. The Effect of Oxygen. *Br. J. Radiol.*, **46**, 450.

MCNALLY, N. J. (1975*a*) A Comparison of the Effects of Radiation on Tumour Growth Delay and Cell Survival. The Effect of Radiation Quality. *Br. J. Radiol.*, **48**, 141.

MCNALLY, N. J. (1975*b*) The Effect of an Hypoxic Cell Sensitizer on Tumour Growth Delay and Cell Survival. *Br. J. Cancer*, **32**, 610.

STEEL, G. G., ADAMS, K. & BARRETT, J. C. (1966) Analysis of the Cell Population Kinetics of Transplanted Tumours of Widely Differing Growth Rate. *Br. J. Cancer*, **20**, 784.

STEPHENS, T. C., PEACOCK, J. H. & STEEL, G. G. (1977) Cell Survival in B16 Melanoma after Treatment with Combinations of Cytotoxic Agents: Lack of Potentiation. *Br. J. Cancer*, **36**, 84.

TWENTYMAN, P. R. (1977) Sensitivity to Cytotoxic Agents of the EMT6 Tumour *In vivo*: Tumour Volume *versus In vitro* Plating. 1. Cyclophosphamide. *Br. J. Cancer*, **35**, 208.

TWENTYMAN, P. R. & BLEEHEN, N. M. (1975) Studies of "Potentially Lethal Damage" in EMT6 Mouse Tumour Cells Treated with Bleomycin either *In vitro* or *In vivo*. *Br. J. Cancer*, **32**, 491.